# Differences in the components of metabolic syndrome by age and sex: a cross-sectional and longitudinal analysis of a cohort of middle-aged and older Japanese adults

**DOI:** 10.1186/s12877-023-04145-0

**Published:** 2023-07-17

**Authors:** Yuji Hiramatsu, Hiroo Ide, Yuji Furui

**Affiliations:** 1 Healthcare Data Science Research Unit, Institute for Future Initiatives, The University of Tokyo, 7-3-1, Hongo, Bunkyo-Ku, Tokyo, 113-0033 Japan; 2 MCVP Division, AXA Life Insurance Co., Ltd, Tokyo, Japan

**Keywords:** Metabolic syndrome, MetS, Sex, Age, Sex difference, Older population, Lifestyle, Cardiovascular disease, Japan

## Abstract

**Background:**

The prevalence of metabolic syndrome (MetS) in Japan, a super-aged society, is increasing and poses a major public health issue. Several studies have reported sex differences in the association between age and MetS prevalence. This study aimed to examine the association between age and the prevalence of MetS based on multiple screening criteria and MetS components by sex.

**Methods:**

We used 6 years of individual-level longitudinal follow-up data (June 2012 to November 2018; checkup year: 2012–2017) of middle-aged and older adults aged 40–75 years in Japan (*N* = 161,735). The Joint Interim Statement criteria, International Diabetes Federation criteria, and another set of criteria excluding central obesity were used as the screening criteria for MetS. The prevalence of MetS and MetS components was cross-sectionally analyzed according to sex and age. A longitudinal association analysis of age, MetS, and MetS components by sex was performed using a multilevel logistic model, adjusted for lifestyle- and regional-related factors.

**Results:**

Sex differences were observed in the prevalence and association of MetS and MetS components. In all age groups, the prevalence of central obesity was higher among women, and the prevalence of high blood pressure and fasting glucose was higher among men (*P* < 0.001). The prevalence of high triglyceride and low high-density lipoprotein cholesterol was higher among women aged > 60 years (*P* < 0.05). Based on the criteria of the Joint Interim Statement and International Diabetes Federation, the prevalence of MetS was higher among women than in men aged > 55 years (*P* < 0.001). Men had a higher prevalence of MetS without central obesity than women in all age groups (*P* < 0.001). The odds ratio for MetS and MetS components with aging was greater among women than in men.

**Conclusions:**

Medical management should be based on the prevalence of MetS and its components according to sex and age. In particular, the high prevalence of MetS without central obesity in middle-aged and older Japanese men suggests that the adoption of the Joint Interim Statement criteria, which do not precondition central obesity, should be considered.

**Supplementary Information:**

The online version contains supplementary material available at 10.1186/s12877-023-04145-0.

## Background

Non-communicable diseases account for over 30% of the national healthcare expenditure in Japan. Among non-communicable diseases, stroke and ischemic heart disease are the leading causes of disability-adjusted life years and death [1]. The prevention of cardiovascular disease (CVD) remains a public health concern in Japan.

Metabolic syndrome (MetS) is a cluster of metabolic abnormalities, including central obesity, high blood pressure, high fasting glucose, and dyslipidemia; it is a risk factor for CVD and type 2 diabetes mellitus [2]. In Japan, the prevalence of MetS has increased in recent years along with that of stroke and ischemic heart disease [3–5]. Furthermore, the prevalence of MetS increases with age, and the prevention of MetS in middle-aged and older populations is an important public health issue in Japan, where the population is rapidly aging [6]. Studies in Asia, Europe, and the United States have shown differences in the association between age and MetS prevalence based on sex [7–16], and some studies in Asia have reported that the prevalence of MetS is higher among women than in men aged > 60 years [12–15]. In addition to sex and age, various other factors, such as lifestyle [12, 15, 17–20], geography [3, 6, 21, 22], race [2, 9, 11, 20], and socio-economic status [8, 9, 11, 18, 23, 24], have been reported to affect the prevalence of MetS and CVD.

Several screening criteria for MetS have been proposed, each focusing on different MetS components. For example, the World Health Organization proposed criteria that emphasize insulin resistance as an upstream factor for CVD [25], while the International Diabetes Federation (IDF) proposed criteria with central obesity as a prerequisite, along with cut-off points that consider sex and race differences [26]. Subsequently, the Joint Interim Statement (JIS) criteria were proposed to standardize the criteria. According to the JIS criteria, the diagnosis of MetS is based on the presence of three or more MetS components and does not consider central obesity a prerequisite [2]. However, in Japan, screening criteria for MetS have adopted central obesity as a prerequisite. Takahara et al. reported that according to the Japanese annual health checkup data, more than half of the participants diagnosed with MetS based on the JIS criteria had three or more MetS components without central obesity in both men and women [27]. This suggests that in addition to MetS caused by central obesity, there are other MetS conditions not involving central obesity [28]. Iso et al. reported a higher risk of CVD in participants with multiple MetS components, even without central obesity [29].

According to previous studies, there are two types of MetS conditions in Japan: those involving central obesity and those not involving it. Both conditions are important risk factors for CVD. However, as the current Japanese MetS criteria are based on the precondition of central obesity, population with MetS without central obesity may be missed during screening. Therefore, it is important to examine the prevalence of MetS using each set of criteria and identify its risk factors to prevent CVD in Japan. Our study aimed to analyze the differences in the prevalence of MetS components between men and women by age group and to examine how the prevalence differs according to the MetS screening criteria adopted: IDF, JIS, and the criteria not considering central obesity at all, to screen population with MetS without central obesity. The primary objective was to cross-sectionally analyze the prevalence of MetS and MetS components by sex and age group in a 6-year population-based cohort of more than 150,000 middle-aged and older Japanese participants. The second objective was to conduct a longitudinal association analysis between age and MetS and MetS components in men and women, with adjustment for lifestyle- and region-related factors.

## Methods

### Study design

This study was conducted using 6-year longitudinal follow-up data of a Japanese population-based cohort. The prevalence of MetS based on three different screening criteria and five MetS components was analyzed by sex and age in a middle-aged and older Japanese population. In addition, we conducted a longitudinal analysis to examine the association of age, region, and lifestyle with MetS and MetS components.

### Study participants

The Shizuoka Kokuho Database [30], curated by the Shizuoka Prefecture National Health Insurance (NHI) database in central Japan (Figure S1), was used. The Shizuoka Kokuho Database consists of individual-level longitudinal follow-up data of NHI enrollees: registration information, demographics, results of lifestyle-related interviews, and clinical measurements and examinations performed at annual preventive medical checkups for individuals aged 40–75 years. Of the recorded clinical measurements and findings, the following measures included in MetS screening were used: waist circumference (WC), systolic and diastolic blood pressure, triglycerides, high-density lipoprotein cholesterol (HDL-C), fasting plasma glucose, and hemoglobin A1c. Of the individuals enrolled in the NHI from June 2012 to November 2018 (checkup year: 2012–2017), those aged 40–70 years in 2012 (45–75 years in 2017) with availability of clinical measurements and records were included in the analysis (men: 69,643; women: 92,092).

### Outcomes and variables

#### Definition of MetS

Table 1 presents the criteria for MetS components used in MetS screening: central obesity, high blood pressure, high triglyceride level, low HDL-C level, and high fasting glucose level. The population-based cut-off point for WC in Asia [2] was used as the criterion for central obesity, and the following three MetS screening criteria were used: JIS [2]; IDF [26]; and a criterion without central obesity (referred to as not-involving waist circumference (NWC) in this study). JIS or similar criteria have been used in several Asian studies [12–16]. The JIS criteria defines MetS as the presence of three or more of five components. The IDF criteria define MetS as the presence of two or more of the remaining four components, with central obesity as a prerequisite. The NWC criteria designed for this study is intended to complement the IDF definition of MetS and define MetS as the presence of three or more of the remaining four components without central obesity. In Japan, a higher risk of CVD has been reported in a population without central obesity but with other MetS components [7]. The NWC criteria have been designed to screen the population without central obesity that may have been missed in the Japanese MetS screening. As indicated by the definition, the prevalence of MetS based on the JIS criteria is the sum of its prevalence based on the IDF and NWC criteria.

**Table 1:** Criteria for screening of metabolic syndrome

MetS Components	Criteria
(1) Central obesity^†^	
	Men: WC ≥ 90 cm
	Women: WC ≥ 80 cm
(2) High blood pressure	
	Systolic/diastolic blood pressure ≥ 130/85 mmHg and/or drug treatment for high blood pressure
(3) High triglycerides	
	Triglycerides ≥ 150 mg/dL and/or drug treatment for high triglycerides
(4) Low HDL-C	
	Men: HDL-C < 40 mg/dL
	Women: HDL-C < 50 mg/dL
(5) High fasting glucose	
	Fasting plasma glucose ≥ 100 mg/dL and/or HbA1c ≥ 5.6% and/or drug treatment for high fasting glucose
MetS	Criteria
JIS	
	Three or more of five MetS components (1–5)
IDF	
	Two or more of four MetS components (2–4), with central obesity as a prerequisite (1)
NWC	
	Three or more of four MetS components (2–4)

*MetS*: metabolic syndrome, *WC* waist circumference, *HDL-C* high-density lipoprotein cholesterol, *HbA1c* hemoglobin A1c, *JIS* Joint Interim Statement, *IDF* International Diabetes Federation, *NWC* not-involving waist circumference

^†^Asian-based criteria

#### Demographic and regional variables

Sex and age at checkup were used as demographic variables. Age was divided into seven groups, ranging from 40 to 75 years, at 5-year intervals. Akahori et al. [31] analyzed regional nutrient intake data from the Shizuoka Prefecture Nutrient Intake Survey [32] and reported regional differences in nutrient intake and health disparities. In addition, several studies have reported a relationship between socio-economic status and MetS, with sex differences [8, 11, 18, 23, 24]. Furthermore, there are reports of an increasing prevalence of hypertension, dyslipidemia, and hyperglycemia in the urban areas of Japan [3, 33]. Considering the results of these studies, we divided the 34 study regions into seven clusters using spatial clustering based on regional variables and geographic adjacencies among the regions. The details of the spatial clustering are described in the statistical analysis section.

#### Lifestyle-related questionnaire

Japanese NHI enrollees are required to complete a lifestyle-related questionnaire designed by the government based on the association of lifestyle [34] with MetS and CVD at the annual checkup. In the present study, excluding the items related to drug treatment, we used the following 11 items of the questionnaire as lifestyle-related questionnaire variables for adjustment in the association analysis: smoking; exercise habits; physical activity; walking speed; eating rate; skipping breakfast; late-night dinner; snacking; alcohol consumption frequency; alcohol consumption; and sleeping. Supplementary Table S1 presents the details of the questionnaire items and response categories. Exercise habits, physical activity, and walking speed were related to energy consumption, whereas eating rate, skipping breakfast, late-night dinner, and snacking were related to dietary habits.

### Statistical analysis

#### Spatial clustering

We performed spatial clustering to cluster the regional variables and used them in the association analysis. For hierarchical clustering [35], considering the regional variables (nutritional intake, number of population and population density, average income, employment rate, and number of hospitals and clinics) [36] and geographic adjacencies, we aggregated the 34 target regions into seven clusters. For nutritional intake, we used the following 11 items associated with MetS components: total energy, protein, fat, carbohydrate, potassium, calcium, zinc, cholesterol, dietary fiber, salt, and unsaturated fatty acid [37–48]. Seven clusters were selected because they showed the best goodness of fit in the likelihood ratio test for the association analysis of MetS with JIS criteria. Furthermore, the Kruskal–Wallis rank sum test was used to test whether the regional variables used for clustering differed among clusters.

#### Descriptive statistics

Descriptive statistics were analyzed cross-sectionally to examine participant characteristics and the prevalence of MetS and MetS components by sex, age group, and checkup year. Demographic variables, regional cluster variables, lifestyle-related variables, and clinical measurements and findings are summarized by checkup year (2012–2017) and sex. Categorical variables are summarized as proportions and continuous variables as means and standard deviations. Categorical variables were tested for sex differences using the chi-square test, and continuous variables were tested using the Wilcoxon rank sum test. The prevalence of MetS and MetS components and the percentage of prevalence of MetS components are summarized by checkup year and sex, and sex differences were tested using the chi-square test. In addition, the prevalence of MetS and MetS components and the number of prevalent MetS components in 2012 and 2017 are summarized by sex and age group, and sex difference was tested using the chi-square test.

#### Association analysis

The association between age and other variables and MetS and MetS components was examined by sex. For the association analysis, 102,127 participants were included, excluding those receiving drug treatment for hypertension, hyperglycemia, and dyslipidemia. The associations between age and lifestyle-related factors and MetS and MetS components were analyzed longitudinally using a generalized linear mixed-effects model. In addition, because MetS and MetS components are binary variables, intraclass correlation coefficients (ICC) at the individual and regional levels were calculated using a random intercept logistic model [49]. Supplementary Table S2 shows the individual- and region-level ICCs for each outcome according to sex. Individual-level ICCs were greater than 0.5 for all outcomes. This was because the differences in outcomes between individuals were large, whereas the differences in outcomes between time points for each individual were small. In contrast, all region-level ICCs were less than 0.1. Based on these ICCs, we conducted an association analysis using a multilevel logistic model with random intercepts at the individual-level. A *P*-value of 0.05 or less was considered to indicate statistical significance in all analyses. All analyses were performed using R version 4.0.2.

## Results

### Descriptive statistics

#### Participants’ characteristics

**Fig. 1:**
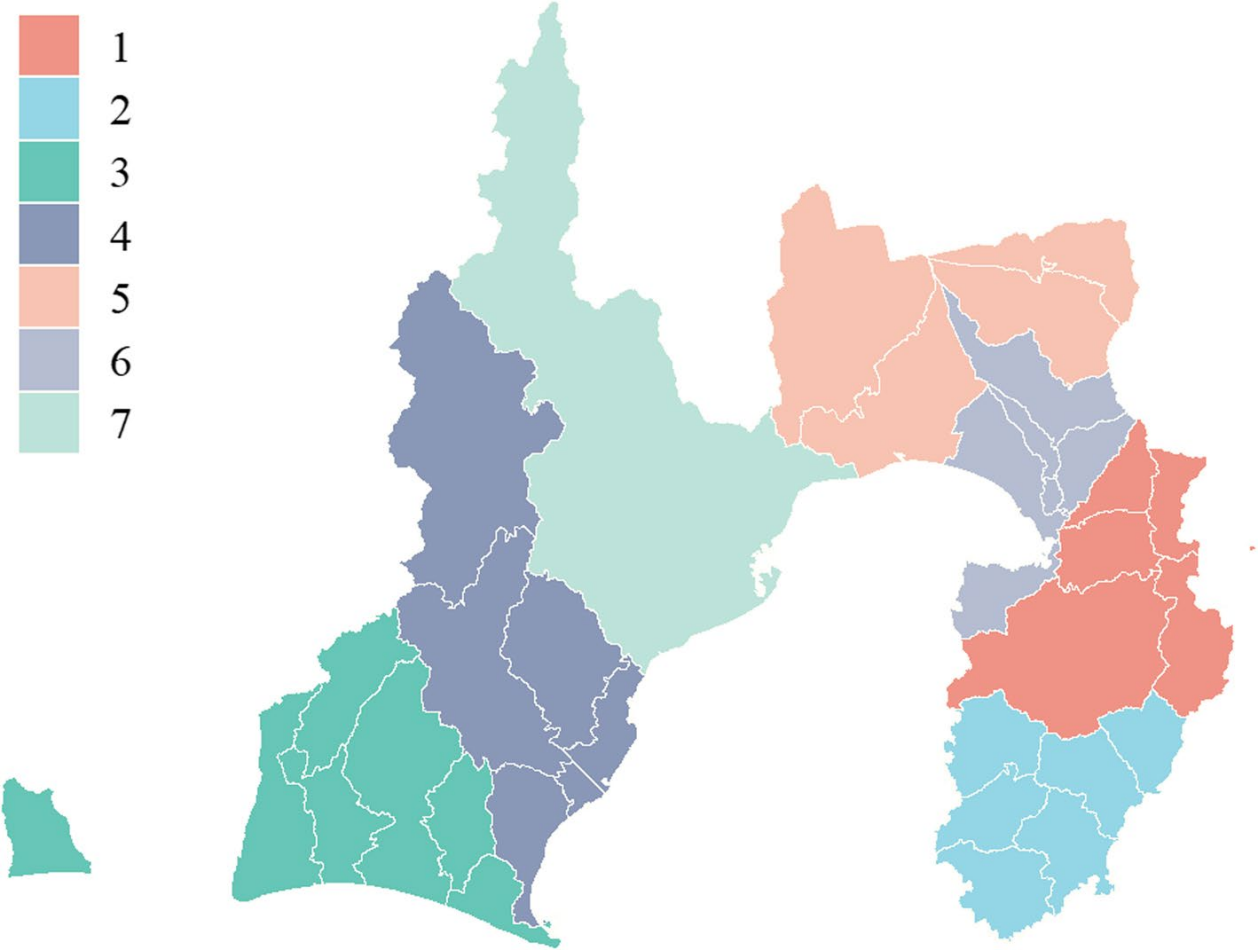
Seven regional clusters by spatial clustering

Figure 1 shows the spatial clustering of the 34 regions into seven regional clusters. The Kruskal–Wallis rank sum test showed that all differences between the clusters of variables used in spatial clustering were significant (*P* < 0.05).

Supplementary Table S3 summarizes the descriptive statistics for age, regional clusters, clinical measurements and examinations, lifestyle-related items by sex and year, and sex differences. The mean age of men was 62.4 years (standard deviation: 6.9) and that of women was 62.8 years (standard deviation: 6.0) in 2012. In all checkup years, the differences between men and women in WC, systolic and diastolic blood pressure, triglyceride, HDL-C, fasting plasma glucose, and hemoglobin A1c levels were significant (*P* < 0.001), with men having higher values. Smoking rates in men were significantly higher than those in women by 4–5 times (*P* < 0.001) but decreased over the years (24.9% in 2012; 20.8% in 2017). In 2012, 2013, and 2015, the differences in exercise habits, physical activity, and walking speed between men and women were significant (*P* < 0.05), and the lifestyle of both sexes tended to improve over the years. In addition, the differences between men and women in dietary habits (eating rate, skipping breakfast, and late-night dinner) and drinking habits (alcohol consumption frequency and alcohol consumption) were significant (*P* < 0.001). Overall, most lifestyle-related factors improved over the years.

#### Prevalence of MetS and MetS components by sex

Table 2 shows the prevalence of MetS (by JIS, IDF, and NWC criteria) and MetS components (central obesity, high blood pressure, high triglyceride, low HDL-C, and high fasting glucose levels) and the number of MetS components by sex and year and the statistical differences between men and women. For all checkup years, there were significant differences in the prevalence of MetS, MetS components, excluding high triglyceride levels in 2016 and 2017, and the number of MetS components between the sexes (*P* < 0.001). Among the MetS components, the prevalence of central obesity and low HDL-C levels was significantly higher in women, whereas the prevalence of other components was significantly higher in men. Additionally, the prevalence of three or more components, including central obesity, was higher in women. In comparison, the prevalence of three or more components, excluding central obesity, was higher in men. In most checkup years, of all MetS components, high blood pressure was most common among men and high fasting glucose levels were most common among women.

**Table 2:** Prevalence of MetS and MetS components by sex and year of checkup

	**2012**					**2013**					**2014**				
	**Men (*N* = 69,643)**		**Women (*N* = 92,092)**		***P*-value**^**†**^	**Men**		**Women**		***P*-value**^**†**^	**Men**		**Women**		***P*-value**^**†**^
Central obesity	22.7%		49.6%		< 0.001	23.1%		49.7%		< 0.001	23.2%		50.2%		< 0.001
High blood pressure	63.0%		52.1%		< 0.001	64.1%		53.5%		< 0.001	65.8%		54.8%		< 0.001
High triglyceride level	38.3%		33.5%		< 0.001	38.8%		35.8%		< 0.001	39.0%		37.0%		< 0.001
Low HDL-C level	10.0%		12.9%		< 0.001	10.1%		13.0%		< 0.001	9.9%		12.4%		< 0.001
High fasting glucose level	51.7%		45.0%		< 0.001	59.0%		54.9%		< 0.001	64.1%		60.4%		< 0.001
Number of MetS components					< 0.001					< 0.001					< 0.001
0	13.7%		15.6%			11.4%		12.7%			9.6%		10.9%		
1	28.0%		25.5%			27.1%		24.3%			26.2%		23.6%		
2	29.1%		25.7%			29.8%		25.9%			30.6%		26.0%		
3	19.0%		19.2%			20.4%		21.0%			21.6%		22.2%		
4	8.6%		11.1%			9.6%		12.7%			9.9%		13.7%		
5	1.6%		2.8%			1.8%		3.4%			2.0%		3.5%		
Number of MetS components except central obesity					< 0.001					< 0.001					< 0.001
0	14.8%		22.3%			12.4%		17.7%			10.5%		15.3%		
1	31.7%		33.5%			30.3%		32.6%			29.4%		31.8%		
2	32.3%		26.3%			33.7%		28.5%			34.9%		30.2%		
3	17.9%		14.6%			20.0%		17.0%			21.3%		18.3%		
4	3.2%		3.5%			3.6%		4.2%			3.9%		4.4%		
JIS	29.2%		33.1%		< 0.001	31.8%		37.1%		< 0.001	33.5%		39.5%		< 0.001
IDF	16.7%		28.4%		< 0.001	17.8%		31.3%		< 0.001	18.3%		33.2%		< 0.001
NWC	12.5%		4.8%		< 0.001	14.0%		5.8%		< 0.001	15.2%		6.3%		< 0.001
IDF/JIS	57.2%		85.8%			56.0%		84.4%			54.6%		84.1%		
NWC/JIS	42.8%		14.2%			44.0%		15.6%			45.4%		15.9%		
	**2015**					**2016**					**2017**				
	**Men**	**Women**		***P*-value**^**†**^		**Men**	**Women**		***P*-value**^**†**^		**Men**	**Women**		***P*-value**^**†**^	
Central obesity	23.7%	50.3%		< 0.001		24.5%	50.9%		< 0.001		25.1%	51.6%		< 0.001	
High blood pressure	66.4%	56.0%		< 0.001		67.1%	56.8%		< 0.001		68.8%	59.0%		< 0.001	
High triglyceride level	39.4%	38.0%		< 0.001		40.0%	39.7%		0.4		41.0%	40.8%		0.6	
Low HDL-C level	10.2%	12.7%		< 0.001		10.0%	12.6%		< 0.001		9.8%	12.2%		< 0.001	
High fasting glucose level	65.6%	62.4%		< 0.001		67.2%	64.0%		< 0.001		65.1%	60.7%		< 0.001	
Number of MetS components				< 0.001					< 0.001					< 0.001	
0	9.2%	10.3%				8.4%	9.5%				8.3%	9.6%			
1	25.6%	22.7%				24.7%	22.0%				24.7%	21.9%			
2	30.4%	26.1%				31.3%	26.1%				31.0%	26.0%			
3	22.3%	22.7%				22.7%	23.4%				22.9%	23.3%			
4	10.6%	14.3%				10.7%	15.0%				10.8%	15.3%			
5	2.0%	3.8%				2.1%	3.9%				2.2%	3.8%			
Number of MetS components, excluding central obesity				< 0.001					< 0.001					< 0.001	
0	10.1%	14.2%				9.2%	13.3%				9.1%	13.5%			
1	28.6%	31.1%				28.1%	30.0%				28.0%	30.2%			
2	35.0%	30.8%				36.0%	31.6%				36.0%	31.2%			
3	22.4%	19.4%				22.6%	20.3%				22.7%	20.4%			
4	4.0%	4.6%				4.1%	4.8%				4.2%	4.7%			
JIS	34.8%	40.8%		< 0.001		35.5%	42.3%		< 0.001		35.9%	42.5%		< 0.001	
IDF	18.9%	34.2%		< 0.001		19.6%	35.3%		< 0.001		20.2%	35.6%		< 0.001	
NWC	15.9%	6.6%		< 0.001		15.9%	7.0%		< 0.001		15.7%	6.9%		< 0.001	
IDF/JIS	54.3%	83.8%				55.2%	83.5%				56.3%	83.8%			
NWC/JIS	45.7%	16.2%				44.8%	16.5%				43.7%	16.2%			

*MetS* metabolic syndrome, *HDL-C* high-density lipoprotein cholesterol, *JIS* Joint Interim Statement, *IDF* International Diabetes Federation, *NWC* not-involving waist circumference

^†^Chi-square test

According to the JIS and IDF criteria, the prevalence of MetS was significantly higher in women in all years, whereas according to the NWC criteria, the prevalence of MetS was significantly higher in men. In all checkup years, the ratio of NWC to JIS was greater than 40% for men but less than 20% for women.

#### Prevalence of MetS and MetS components by sex and age in 2012 and 2017

Supplementary Table S4 shows the prevalence of MetS and MetS components in 2012 and 2017 by sex and age group and the differences between men and women. The prevalence of central obesity was significantly higher (*P* < 0.001) in women across all age groups in both checkup years, whereas in men, the prevalence decreased in the older group. The prevalence of high blood pressure was significantly higher (*P* < 0.001) in men in all age groups in both checkup years and increased with age in both sexes. The prevalence of high triglyceride levels was higher in men in the younger age groups; however, in the 65–70-year age group in 2012 and in the 70–75 age group in 2017, a significantly higher prevalence was observed in women (*P* < 0.05). The prevalence of low HDL-C levels was higher in men in the younger age groups; however, it was significantly higher in women in the older age groups (≥ 60–65 years) in both 2012 and 2017 (*P* < 0.05). The prevalence of high fasting glucose levels was significantly higher in men across all age groups in both checkup years (*P* < 0.001) and increased with age in both sexes. In general, a significantly higher prevalence of components related to dyslipidemia was observed in women in the older age groups.

The prevalence of MetS based on the JIS criteria was significantly higher in women in the older age groups (≥ 60–65 years in 2012 and ≥ 65–70 years in 2017) (*P* < 0.001). The prevalence of MetS based on the IDF criteria was also significantly higher in women in the older age groups (≥ 55–60 years in 2012 and ≥ 60–65 years in 2017) (*P* < 0.001). In contrast, the prevalence of MetS based on the NWC criteria increased with age in both sexes; however, the prevalence in men was more than three times higher than that in women across all age groups (*P* < 0.001).

### Association analysis

Tables 3 and 4 show the association analysis findings of MetS components and MetS in men, and Tables 5 and 6 show the association analysis findings of MetS components and MetS in women, respectively.

**Table 3:** Association analysis for MetS components in men (*N* = 42,690)

		Central obesity		High blood pressure		High triglyceride level		Low HDL-C level		High fasting glucose level	
Variable	Category	ORs (95% CI)	*P-*value	ORs (95% CI)	*P-*value	ORs (95% CI)	*P-*value	ORs (95% CI)	*P-*value	ORs (95% CI)	*P-*value
Age, years	40–45	Reference		Reference		Reference		Reference		Reference	
	45–50	1.318 (0.976–1.778)	.072	1.235 (1.065–1.433)	< 0.001	1.101 (0.933–1.300)	.254	0.946 (0.679–1.318)	.742	2.463 (2.083–2.914)	< 0.001
	50–55	1.332 (0.931–1.904)	.116	2.016 (1.708–2.380)	< 0.001	1.082 (0.893–1.310)	.423	0.845 (0.571–1.252)	.401	4.591 (3.775–5.584)	< 0.001
	55–60	1.079 (0.743–1.568)	.688	3.251 (2.751–3.843)	< 0.001	0.857 (0.704–1.042)	.122	0.914 (0.609–1.371)	.663	7.853 (6.420–9.607)	< 0.001
	60–65	0.996 (0.695–1.428)	.983	5.029 (4.289–5.896)	< 0.001	0.679 (0.563–0.818)	< 0.001	0.712 (0.483–1.051)	.088	11.688 (9.621–14.199)	< 0.001
	65–70	0.956 (0.671–1.362)	.801	6.095 (5.214–7.123)	< 0.001	0.544 (0.453–0.653)	< 0.001	0.714 (0.488–1.045)	.083	20.819 (17.149–25.274)	< 0.001
	70–75	1.085 (0.751–1.566)	.665	7.467 (6.351–8.780)	< 0.001	0.436 (0.360–0.528)	< 0.001	0.866 (0.583–1.287)	.477	37.899 (30.933–46.433)	< 0.001
Smoking^†^	Yes	Reference		Reference		Reference		Reference		Reference	
	No	1.653 (1.386–1.971)	< 0.001	1.204 (1.127–1.287)	< 0.001	0.566 (0.521–0.616)	< 0.001	0.359 (0.300–0.430)	< 0.001	1.061 (0.974–1.157)	.174
Exercise habits^†^	Yes	Reference		Reference		Reference		Reference		Reference	
	No	1.306 (1.163–1.467)	< 0.001	1.145 (1.089–1.205)	< 0.001	1.180 (1.107–1.257)	< 0.001	1.216 (1.062–1.393)	< 0.001	1.120 (1.054–1.190)	< 0.001
Physical activity^†^	Yes	Reference		Reference		Reference		Reference		Reference	
	No	1.357 (1.218–1.512)	< 0.001	1.067 (1.017–1.119)	< 0.001	1.398 (1.318–1.483)	< 0.001	1.318 (1.164–1.491)	< 0.001	1.020 (0.964–1.079)	.500
Walking speed^†^	Yes	Reference		Reference		Reference		Reference		Reference	
	No	1.279 (1.139–1.437)	< 0.001	0.973 (0.925–1.022)	.274	1.028 (0.966–1.094)	.379	1.326 (1.161–1.515)	< 0.001	1.036 (0.975–1.100)	.254
Eating rate^†^	Fast	Reference		Reference		Reference		Reference		Reference	
	Normal	0.654 (0.576–0.743)	< 0.001	0.908 (0.858–0.961)	< 0.001	0.788 (0.734–0.845)	< 0.001	0.880 (0.756–1.024)	.097	0.889 (0.829–0.953)	< 0.001
	Slow	0.478 (0.372–0.615)	< 0.001	0.688 (0.623–0.759)	< 0.001	0.572 (0.504–0.650)	< 0.001	0.788 (0.598–1.038)	.090	0.737 (0.654–0.832)	< 0.001
Skipping breakfast^†^	Yes	Reference		Reference		Reference		Reference		Reference	
	No	0.819 (0.676–0.992)	.041	0.815 (0.747–0.888)	< 0.001	0.795 (0.716–0.883)	< 0.001	0.815 (0.653–1.016)	.069	1.062 (0.956–1.180)	.259
Late-night dinner^†^	Yes	Reference		Reference		Reference		Reference		Reference	
	No	0.774 (0.673–0.891)	< 0.001	0.852 (0.798–0.908)	< 0.001	0.924 (0.855–0.999)	.048	0.967 (0.816–1.145)	.695	0.834 (0.773–0.901)	< 0.001
Snacking^†^	Yes	Reference		Reference		Reference		Reference		Reference	
	No	0.768 (0.661–0.893)	< 0.001	1.070 (0.996–1.149)	.063	0.949 (0.870–1.034)	.232	1.175 (0.982–1.406)	.079	0.853 (0.785–0.928)	< 0.001
Alcohol consumption frequency^†^	Every day	Reference		Reference		Reference		Reference		Reference	
	Sometimes	0.921 (0.794–1.070)	.283	0.555 (0.521–0.591)	< 0.001	0.972 (0.898–1.053)	.490	2.205 (1.836–2.648)	< 0.001	1.256 (1.163–1.357)	< 0.001
	Rarely	1.173 (0.968–1.422)	.104	0.526 (0.486–0.570)	< 0.001	1.179 (1.066–1.304)	< 0.001	3.503 (2.815–4.360)	< 0.001	1.403 (1.271–1.549)	< 0.001
Alcohol consumption^†^	< 180 mL	Reference		Reference		Reference		Reference		Reference	
	180–360 mL	0.955 (0.829–1.101)	.529	1.261 (1.187–1.341)	< 0.001	1.087 (1.006–1.174)	.034	0.799 (0.677–0.943)	< 0.001	0.997 (0.927–1.072)	.934
	360–540 mL	1.157 (0.965–1.387)	.116	1.617 (1.494–1.750)	< 0.001	1.542 (1.398–1.701)	< 0.001	0.679 (0.538–0.859)	< 0.001	1.077 (0.978–1.185)	.131
	> 540 mL	1.154 (0.870–1.530)	.320	2.067 (1.824–2.343)	< 0.001	2.122 (1.825–2.468)	< 0.001	0.726 (0.501–1.051)	.090	1.125 (0.966–1.310)	.130
Sleeping^†^	Yes	Reference		Reference		Reference		Reference		Reference	
	No	1.108 (0.978–1.256)	.108	0.867 (0.820–0.916)	< 0.001	0.856 (0.799–0.916)	< 0.001	0.889 (0.768–1.028)	.112	1.058 (0.990–1.130)	.096
Regional cluster	1	Reference		Reference		Reference		Reference		Reference	
	2	0.803 (0.504–1.279)	.356	3.703 (3.124–4.389)	< 0.001	1.382 (1.109–1.721)	< 0.001	0.919 (0.542–1.560)	.755	0.391 (0.310–0.493)	< 0.001
	3	0.653 (0.475–0.896)	< 0.001	0.712 (0.636–0.797)	< 0.001	0.523 (0.451–0.607)	< 0.001	0.882 (0.615–1.264)	.494	2.390 (2.048–2.790)	< 0.001
	4	0.613 (0.441–0.853)	< 0.001	1.254 (1.116–1.409)	< 0.001	0.577 (0.495–0.673)	< 0.001	0.833 (0.574–1.209)	.336	2.654 (2.260–3.116)	< 0.001
	5	1.123 (0.819–1.539)	.472	1.437 (1.279–1.614)	< 0.001	0.824 (0.707–0.961)	.014	1.048 (0.728–1.509)	.800	1.193 (1.017–1.399)	.031
	6	1.070 (0.701–1.633)	.755	1.375 (1.176–1.609)	< 0.001	0.906 (0.738–1.113)	.349	1.006 (0.614–1.648)	.980	1.033 (0.834–1.280)	.763
	7	1.033 (0.760–1.406)	.834	1.648 (1.472–1.846)	< 0.001	0.649 (0.559–0.753)	< 0.001	0.981 (0.685–1.404)	.916	2.532 (2.167–2.959)	< 0.001

*MetS* metabolic syndrome, *HDL-C* high-density lipoprotein cholesterol

^†^See Supplementary Table S1 for the definitions of each lifestyle-related variables

**Table 4:** Association analysis for the JIS, IDF, and NWC criteria for MetS in men (*N* = 42,690)

		JIS		IDF		NWC	
Variable	Category	ORs (95% CI)	*P-*value	ORs (95% CI)	*P-*value	ORs (95% CI)	*P-*value
Age, years	40–45	Reference		Reference		Reference	
	45–50	1.914 (1.479–2.476)	< 0.001	2.130 (1.537–2.951)	< 0.001	1.337 (0.990–1.806)	.059
	50–55	2.317 (1.716–3.129)	< 0.001	2.292 (1.556–3.375)	< 0.001	1.579 (1.116–2.233)	< 0.001
	55–60	2.172 (1.592–2.964)	< 0.001	1.677 (1.115–2.522)	.013	1.751 (1.228–2.495)	< 0.001
	60–65	2.102 (1.556–2.838)	< 0.001	1.620 (1.092–2.405)	.017	1.771 (1.259–2.491)	< 0.001
	65–70	2.339 (1.740–3.144)	< 0.001	1.790 (1.214–2.638)	< 0.001	1.948 (1.392–2.725)	< 0.001
	70–75	3.053 (2.252–4.141)	< 0.001	2.411 (1.616–3.598)	< 0.001	2.333 (1.651–3.295)	< 0.001
Smoking^†^	Yes	Reference		Reference		Reference	
	No	0.917 (0.808–1.041)	.181	1.202 (1.007–1.434)	.042	0.801 (0.698–0.920)	< 0.001
Exercise habits^†^	Yes	Reference		Reference		Reference	
	No	1.226 (1.122–1.339)	< 0.001	1.316 (1.163–1.489)	< 0.001	1.173 (1.062–1.296)	< 0.001
Physical activity^†^	Yes	Reference		Reference		Reference	
	No	1.363 (1.257–1.479)	< 0.001	1.373 (1.226–1.538)	< 0.001	1.312 (1.196–1.439)	< 0.001
Walking speed^†^	Yes	Reference		Reference		Reference	
	No	1.229 (1.126–1.341)	< 0.001	1.363 (1.207–1.539)	< 0.001	1.092 (0.989–1.206)	.081
Eating rate^†^	Fast	Reference		Reference		Reference	
	Normal	0.722 (0.654–0.797)	< 0.001	0.677 (0.593–0.772)	< 0.001	0.795 (0.711–0.888)	< 0.001
	Slow	0.486 (0.403–0.586)	< 0.001	0.441 (0.335–0.579)	< 0.001	0.657 (0.534–0.809)	< 0.001
Skipping breakfast^†^	Yes	Reference		Reference		Reference	
	No	0.847 (0.729–0.983)	.029	0.869 (0.716–1.055)	.157	0.828 (0.699–0.979)	.028
Late-night dinner^†^	Yes	Reference		Reference		Reference	
	No	0.778 (0.699–0.867)	< 0.001	0.752 (0.652–0.868)	< 0.001	0.814 (0.720–0.920)	< 0.001
Snacking^†^	Yes	Reference		Reference		Reference	
	No	0.869 (0.771–0.979)	.021	0.769 (0.658–0.900)	< 0.001	0.947 (0.825–1.086)	.433
Alcohol consumption frequency^†^	Every day	Reference		Reference		Reference	
	Sometimes	0.921 (0.822–1.032)	.157	0.792 (0.677–0.927)	< 0.001	1.003 (0.881–1.141)	.969
	Rarely	1.224 (1.057–1.417)	< 0.001	1.058 (0.867–1.291)	.577	1.382 (1.173–1.628)	< 0.001
Alcohol consumption^†^	< 180 mL	Reference		Reference		Reference	
	180–360 mL	0.947 (0.850–1.054)	.318	0.847 (0.729–0.985)	.031	1.007 (0.890–1.138)	.917
	360–540 mL	1.101 (0.957–1.267)	.177	0.975 (0.804–1.182)	.794	1.195 (1.020–1.401)	.027
	> 540 mL	1.255 (1.011–1.558)	.039	1.038 (0.776–1.387)	.803	1.360 (1.069–1.731)	.012
Sleeping^†^	Yes	Reference		Reference		Reference	
	No	0.965 (0.876–1.063)	.475	1.037 (0.908–1.184)	.593	0.950 (0.851–1.061)	.365
Regional cluster	1	Reference		Reference		Reference	
	2	1.117 (0.781–1.596)	.545	0.929 (0.579–1.489)	.760	1.058 (0.723–1.548)	.772
	3	0.731 (0.575–0.929)	.011	0.705 (0.506–0.981)	.038	0.785 (0.604–1.021)	.071
	4	0.875 (0.683–1.120)	.289	0.732 (0.522–1.028)	.072	0.966 (0.739–1.263)	.803
	5	1.163 (0.910–1.487)	.228	1.150 (0.829–1.594)	.403	1.083 (0.829–1.413)	.560
	6	1.183 (0.852–1.643)	.315	1.127 (0.729–1.741)	.590	1.080 (0.756–1.544)	.672
	7	1.232 (0.972–1.563)	.085	1.154 (0.840–1.586)	.375	1.156 (0.893–1.497)	.270

*MetS* metabolic syndrome, *JIS* Joint Interim Statement, *IDF* International Diabetes Federation, *NWC* not-involving waist circumference

^†^See Supplementary Table S1 for the definitions of each lifestyle-related variables

**Table 5:** Association analysis for MetS components in women (*N* = 59,437)

		Central obesity		High blood pressure		High triglycerides		Low HDL-C		High fasting glucose	
Variable	Category	ORs (95% CI)	*P-*value	ORs (95% CI)	*P-*value	ORs (95% CI)	*P-*value	ORs (95% CI)	*P-*value	ORs (95% CI)	*P-*value
Age, years	40–45	Reference		Reference		Reference		Reference		Reference	
	45–50	1.860 (1.508–2.294)	< 0.001	1.705 (1.399–2.078)	< 0.001	1.427 (1.001–2.035)	.050	0.703 (0.478–1.034)	.073	2.342 (1.921–2.856)	< 0.001
	50–55	2.611 (2.056–3.316)	< 0.001	3.409 (2.760–4.210)	< 0.001	1.984 (1.328–2.962)	< 0.001	0.620 (0.398–0.966)	.034	5.707 (4.591–7.096)	< 0.001
	55–60	4.164 (3.280–5.288)	< 0.001	6.614 (5.379–8.133)	< 0.001	2.423 (1.623–3.616)	< 0.001	0.672 (0.433–1.040)	.075	12.538 (10.097–15.569)	< 0.001
	60–65	5.878 (4.659–7.417)	< 0.001	11.737 (9.598–14.352)	< 0.001	2.717 (1.838–4.017)	< 0.001	0.752 (0.494–1.143)	.182	21.721 (17.567–26.857)	< 0.001
	65–70	7.155 (5.676–9.020)	< 0.001	18.739 (15.334–22.899)	< 0.001	2.634 (1.784–3.890)	< 0.001	0.743 (0.490–1.126)	.162	36.285 (29.324–44.899)	< 0.001
	70–75	8.156 (6.426–10.353)	< 0.001	26.265 (21.383–32.262)	< 0.001	2.646 (1.776–3.944)	< 0.001	0.701 (0.456–1.079)	.106	61.787 (49.559–77.031)	< 0.001
Smoking^†^	Yes	Reference		Reference		Reference		Reference		Reference	
	No	1.219 (1.040–1.429)	.015	1.227 (1.095–1.376)	< 0.001	0.489 (0.398–0.602)	< 0.001	0.441 (0.335–0.580)	< 0.001	1.208 (1.059–1.379)	< 0.001
Exercise habits^†^	Yes	Reference		Reference		Reference		Reference		Reference	
	No	1.130 (1.064–1.200)	< 0.001	1.124 (1.072–1.179)	< 0.001	1.086 (0.992–1.188)	.074	1.156 (1.028–1.299)	.015	1.052 (0.997–1.109)	.062
Physical activity^†^	Yes	Reference		Reference		Reference		Reference		Reference	
	No	1.247 (1.181–1.318)	< 0.001	1.008 (0.964–1.053)	.737	1.269 (1.168–1.379)	< 0.001	1.308 (1.176–1.455)	< 0.001	0.980 (0.934–1.029)	.416
Walking speed^†^	Yes	Reference		Reference		Reference		Reference		Reference	
	No	1.388 (1.305–1.475)	< 0.001	1.089 (1.039–1.143)	< 0.001	1.130 (1.033–1.236)	< 0.001	1.094 (0.972–1.231)	.138	0.987 (0.936–1.041)	.631
Eating rate^†^	Fast	Reference		Reference		Reference		Reference		Reference	
	Normal	0.502 (0.464–0.543)	< 0.001	1.034 (0.975–1.098)	.262	0.899 (0.803–1.006)	.063	0.900 (0.776–1.044)	.164	0.874 (0.817–0.934)	< 0.001
	Slow	0.305 (0.268–0.347)	< 0.001	0.799 (0.724–0.880)	< 0.001	0.841 (0.696–1.017)	.074	0.901 (0.705–1.150)	.401	0.756 (0.678–0.844)	< 0.001
Skipping breakfast^†^	Yes	Reference		Reference		Reference		Reference		Reference	
	No	0.806 (0.709–0.916)	< 0.001	0.958 (0.868–1.058)	.396	0.841 (0.706–1.003)	.053	1.016 (0.801–1.290)	.894	1.243 (1.112–1.389)	< 0.001
Late–night dinner^†^	Yes	Reference		Reference		Reference		Reference		Reference	
	No	0.860 (0.776–0.953)	< 0.001	0.938 (0.865–1.018)	.125	0.822 (0.712–0.949)	< 0.001	1.027 (0.840–1.256)	.795	0.891 (0.815–0.975)	.012
Snacking^†^	Yes	Reference		Reference		Reference		Reference		Reference	
	No	0.798 (0.735–0.867)	< 0.001	1.005 (0.940–1.074)	.891	0.949 (0.842–1.071)	.399	0.999 (0.853–1.170)	.992	0.909 (0.846–0.978)	.010
Alcohol consumption frequency^†^	Every day	Reference		Reference		Reference		Reference		Reference	
	Sometimes	1.098 (0.986–1.223)	.089	0.709 (0.653–0.770)	< 0.001	1.141 (0.971–1.342)	.110	1.766 (1.376–2.266)	< 0.001	1.404 (1.280–1.540)	< 0.001
	Rarely	1.015 (0.903–1.141)	.808	0.815 (0.747–0.888)	< 0.001	1.322 (1.110–1.574)	< 0.001	2.727 (2.096–3.549)	< 0.001	1.378 (1.248–1.522)	< 0.001
Alcohol consumption^†^	< 180 mL	Reference		Reference		Reference		Reference		Reference	
	180–360 mL	1.144 (1.039–1.260)	< 0.001	1.132 (1.048–1.223)	< 0.001	1.062 (0.918–1.230)	.417	0.742 (0.599–0.921)	< 0.001	0.882 (0.810–0.960)	< 0.001
	360–540 mL	1.402 (1.166–1.687)	< 0.001	1.734 (1.500–2.006)	< 0.001	1.617 (1.252–2.089)	< 0.001	0.854 (0.568–1.286)	.450	0.884 (0.751–1.041)	.139
	> 540 mL	1.543 (1.091–2.184)	.014	1.901 (1.451–2.489)	< 0.001	1.302 (0.821–2.066)	.263	0.700 (0.326–1.505)	.361	0.671 (0.494–0.910)	.010
Sleeping^†^	Yes	Reference		Reference		Reference		Reference		Reference	
	No	0.930 (0.875–0.990)	.023	0.928 (0.884–0.975)	< 0.001	0.853 (0.777–0.936)	< 0.001	1.031 (0.917–1.159)	.610	1.137 (1.077–1.200)	< 0.001
Regional cluster	1	Reference		Reference		Reference		Reference		Reference	
	2	1.321 (1.048–1.664)	.018	2.774 (2.388–3.222)	< 0.001	1.239 (0.908–1.690)	.177	0.742 (0.476–1.158)	.189	0.307 (0.255–0.370)	< 0.001
	3	1.145 (0.970–1.352)	.110	0.967 (0.868–1.078)	.549	0.659 (0.522–0.832)	< 0.001	0.878 (0.642–1.201)	.416	1.749 (1.534–1.995)	< 0.001
	4	0.332 (0.281–0.393)	< 0.001	1.082 (0.971–1.205)	.152	0.591 (0.468–0.747)	< 0.001	0.869 (0.638–1.184)	.373	2.169 (1.902–2.475)	< 0.001
	5	1.769 (1.501–2.085)	< 0.001	1.187 (1.067–1.321)	< 0.001	0.878 (0.701–1.100)	.258	0.969 (0.714–1.316)	.839	1.007 (0.884–1.146)	.919
	6	1.124 (0.909–1.390)	.279	1.040 (0.906–1.194)	.575	0.911 (0.680–1.221)	.532	0.805 (0.533–1.215)	.302	0.655 (0.553–0.775)	< 0.001
	7	1.032 (0.879–1.212)	.699	1.217 (1.096–1.351)	< 0.001	0.732 (0.584–0.917)	< 0.001	0.930 (0.684–1.263)	.641	2.225 (1.958–2.529)	< 0.001

*MetS* metabolic syndrome, *HDL-C* high-density lipoprotein cholesterol

^†^See Supplementary Table S1 for the definitions of each lifestyle-related variables

**Table 6:** Association analysis for JIS, IDF, and NWC criteria for MetS in women (*N* = 59,437)

		JIS		IDF		NWC	
Variable	Category	ORs (95% CI)	*P-*value	ORs (95% CI)	*P-*value	ORs (95% CI)	*P-*value
Age, years	40–45	Reference		Reference		Reference	
	45–50	1.795 (1.201–2.683)	< 0.001	1.855 (1.222–2.816)	< 0.001	1.865 (0.982–3.543)	.057
	50–55	2.825 (1.820–4.384)	< 0.001	3.027 (1.911–4.794)	< 0.001	2.472 (1.202–5.083)	.014
	55–60	4.131 (2.673–6.383)	< 0.001	3.818 (2.417–6.031)	< 0.001	3.574 (1.749–7.304)	< 0.001
	60–65	6.071 (3.961–9.306)	< 0.001	5.561 (3.551–8.709)	< 0.001	4.628 (2.293–9.340)	< 0.001
	65–70	9.013 (5.888–13.797)	< 0.001	8.021 (5.129–12.542)	< 0.001	6.314 (3.137–12.709)	< 0.001
	70–75	13.274 (8.626–20.428)	< 0.001	11.702 (7.444–18.397)	< 0.001	8.817 (4.353–17.861)	< 0.001
Smoking^†^	Yes	Reference		Reference		Reference	
	No	0.750 (0.611–0.921)	< 0.001	0.840 (0.678–1.041)	.112	0.696 (0.520–0.932)	.015
Exercise habits^†^	Yes	Reference		Reference		Reference	
	No	1.257 (1.163–1.359)	< 0.001	1.250 (1.153–1.356)	< 0.001	1.194 (1.061–1.343)	< 0.001
Physical activity^†^	Yes	Reference		Reference		Reference	
	No	1.109 (1.032–1.191)	< 0.001	1.126 (1.045–1.213)	< 0.001	1.224 (1.098–1.364)	< 0.001
Walking speed^†^	Yes	Reference		Reference		Reference	
	No	1.197 (1.106–1.296)	< 0.001	1.192 (1.098–1.294)	< 0.001	1.124 (0.998–1.265)	.054
Eating rate^†^	Fast	Reference		Reference		Reference	
	Normal	0.817 (0.739–0.902)	< 0.001	0.776 (0.700–0.860)	< 0.001	0.975 (0.840–1.132)	.741
	Slow	0.668 (0.564–0.790)	< 0.001	0.644 (0.541–0.768)	< 0.001	0.792 (0.610–1.029)	.080
Skipping breakfast^†^	Yes	Reference		Reference		Reference	
	No	0.911 (0.771–1.077)	.275	0.887 (0.746–1.056)	.178	1.007 (0.787–1.288)	.956
Late-night dinner^†^	Yes	Reference		Reference		Reference	
	No	0.899 (0.789–1.024)	.110	0.902 (0.787–1.033)	.136	0.800 (0.657–0.974)	.026
Snacking^†^	Yes	Reference		Reference		Reference	
	No	0.901 (0.810–1.003)	.057	0.887 (0.794–0.992)	.035	0.859 (0.732–1.007)	.062
Alcohol consumption frequency^†^	Every day	Reference		Reference		Reference	
	Sometimes	1.033 (0.894–1.193)	.659	0.988 (0.850–1.147)	.873	1.289 (1.017–1.633)	.036
	Rarely	1.220 (1.044–1.424)	.012	1.126 (0.959–1.322)	.147	1.676 (1.305–2.151)	< 0.001
Alcohol consumption^†^	< 180 mL	Reference		Reference		Reference	
	180–360 mL	0.995 (0.875–1.132)	.940	1.017 (0.889–1.162)	.809	0.960 (0.781–1.181)	.701
	360–540 mL	1.249 (0.981–1.589)	.071	1.179 (0.918–1.514)	.196	1.431 (0.982–2.086)	.062
	> 540 mL	1.019 (0.639–1.626)	.936	1.044 (0.641–1.700)	.861	0.796 (0.382–1.661)	.544
Sleeping^†^	Yes	Reference		Reference		Reference	
	No	0.973 (0.898–1.054)	.496	0.982 (0.904–1.068)	.672	0.910 (0.806–1.028)	.128
Regional cluster	1	Reference		Reference		Reference	
	2	0.973 (0.718–1.321)	.863	1.009 (0.738–1.379)	.954	0.864 (0.566–1.319)	.497
	3	0.959 (0.772–1.191)	.704	0.975 (0.780–1.220)	.826	0.858 (0.631–1.167)	.330
	4	0.813 (0.655–1.010)	.061	0.800 (0.640–1.001)	.051	0.853 (0.629–1.156)	.305
	5	1.150 (0.929–1.424)	.199	1.186 (0.953–1.478)	.127	0.955 (0.707–1.290)	.764
	6	0.864 (0.654–1.142)	.304	0.888 (0.666–1.184)	.418	0.806 (0.539–1.207)	.296
	7	1.122 (0.911–1.381)	.279	1.152 (0.930–1.428)	.195	0.990 (0.735–1.333)	.946

*MetS* metabolic syndrome, *JIS* Joint Interim Statement, *IDF* International Diabetes Federation; *NWC* not-involving waist circumference

^†^See Supplementary Table S1 for the definitions of each lifestyle-related variables

#### Central obesity

The odds ratio (OR) for central obesity increased significantly with age in women (*P* < 0.001), whereas no significant associations were found in men. Among the lifestyle-related questionnaire items, the eating rate showed the largest OR. Slower eating was significantly associated with a decrease in the OR for central obesity in both men and women, and a dose–response relationship was found.

#### High blood pressure

The OR for high blood pressure increased significantly with age (*P* < 0.001) in both sexes, with women showing a greater OR. Among lifestyle-related questionnaire items, those related to alcohol consumption showed a large OR; greater frequency and quantity of alcohol consumption were significantly associated with an increase in the OR (*P* < 0.001), and a dose–response relationship was found for alcohol consumption.

#### High triglyceride level

Among men, the OR for a high triglyceride level decreased significantly with age for those aged ≥ 60 years (*P* < 0.001). In contrast, the OR tended to increase significantly with age in women (*P* < 0.05). Among the lifestyle-related questionnaire items, smoking, eating rate, and alcohol consumption showed greater ORs for men, while smoking showed a greater OR for women. For both men and women, smoking was significantly associated with an increased OR (*P* < 0.001), and physical inactivity was significantly associated with an increased OR (*P* < 0.001). In addition, slower eating was significantly associated with a decrease in OR (*P* < 0.001), and a dose–response relationship was found in men. Furthermore, higher alcohol consumption was significantly associated with an increase in the OR (*P* < 0.001), and a dose–response relationship was observed among men.

#### Low HDL-C level

No significant OR for low HDL-C levels was found for age in almost all age groups, for men or women. Among lifestyle-related questionnaires, the alcohol consumption frequency showed the highest OR, and a lower frequency was significantly associated with an increased OR (*P* < 0.001) for both men and women. Moderate alcohol consumption (men: 180–540 mL; women: 180–360 mL) was significantly associated with a decrease in the OR (*P* < 0.001).

#### High fasting glucose level

The OR for a high fasting glucose level increased significantly with age (*P* < 0.001) for both men and women. High fasting glucose levels showed the largest increase in OR among all MetS components. Among the lifestyle-related questionnaire items, the frequencies of eating and alcohol consumption were associated with higher ORs. Slower eating was significantly associated with lower ORs (*P* < 0.001), and a dose–response relationship was found in both men and women. A lower frequency of alcohol consumption was significantly associated with higher ORs (*P* < 0.001) in both men and women.

#### JIS, IDF, and NWC criteria

In men, the OR for MetS prevalence based on the NWC criteria increased significantly with age (*P* < 0.001), and in women, the ORs for MetS prevalence based on the JIS, IDF, and NWC criteria increased significantly (*P* < 0.001), with greater increase in the OR among women with age. These associations were consistent with the results of prevalence by age group in the descriptive statistical analysis. Smoking was significantly associated with a decrease in the OR for MetS prevalence based on IDF criteria (*P* < 0.05) and an increase in the OR for MetS prevalence based on NWC criteria (*P* < 0.001) in men. In contrast, in women, it was significantly associated with an increase in the ORs for MetS prevalence based on JIS and NWC criteria (*P* < 0.05). Lower exercise habits and physical activity were significantly associated with increased OR for MetS based on all criteria in both men and women (*P* < 0.001). In addition, slower eating was significantly associated with a decrease in the OR for MetS based on all criteria among men and the OR for MetS based on the JIS and IDF criteria among women; a dose–response relationship was found (*P* < 0.001). Furthermore, in men, higher alcohol consumption (> 360 mL) was significantly associated with an increase in the OR for MetS prevalence based on NWC criteria, and a dose–response relationship was observed (*P* < 0.05). In contrast, in women, a lower frequency of alcohol consumption was significantly associated with a higher OR for MetS prevalence based on NWC criteria, and a dose–response relationship was observed (*P* < 0.05). Overall, eating rate had the largest OR among the lifestyle-related questionnaire items.

## Discussion

In this study, we analyzed the prevalence and associations of MetS based on three screening criteria and MetS components by sex and age in a 6-year population-based cohort of middle-aged and older Japanese individuals to examine the prevalence of MetS with and without central obesity. Sex differences were found in the prevalence and associations of MetS components and MetS, and the increase in OR with age was larger in women than in men. In addition, men had higher prevalence of MetS conditions without central obesity than did women. Studies on the relationship between sex and age and the prevalence of MetS and MetS components using multiple screening criteria have been conducted in Asian countries, including Japan; however, these were cross-sectional studies [10, 12, 13, 15, 16]. To the best of our knowledge, few studies have examined the prevalence of MetS and MetS components longitudinally in a large cohort of more than 150,000 middle-aged and older adults aged 40–75 years, as in this study. Another strength of our study is that it not only examined prevalence by age but also used a multilevel logistic model adjusted for lifestyle-related variables to examine the significance of the effect of aging more rigorously in a longitudinal setting.

### Sex difference in MetS components

In our study, the prevalence of central obesity was significantly higher among women in all age groups, and the increase in OR with age was significant only for women. Lee et al. reported that the prevalence of central obesity was significantly higher in women using an Asian-based cut-off point for 103,763 Korean men and women aged 66 years in 2008 [10]. Another Korean cross-sectional study reported that among 5,760 men and women in 2010, the prevalence of central obesity was higher in women aged ≥ 40 years, and the prevalence in women was more than twice that in men aged ≥ 70 years [12]. Arai et al. also reported that the prevalence of central obesity was higher in women aged ≥ 50 years among 2,366 Japanese men and women in 2000 [16]. The results of these studies are consistent with our findings. The higher prevalence of central obesity in women with age may be one reason for the significantly increased prevalence of MetS diagnosed based on IDF and JIS criteria in women compared to that in men. The higher prevalence of central obesity among women may be attributable to abdominal obesity caused by hormonal homeostatic dysregulation after menopause in women [8].

The prevalence of MetS components related to dyslipidemia was significantly higher in women aged 65–75 years, who had high triglyceride levels, and those aged 60–75 years, who had low HDL-C levels. In addition, aging had a significant decreasing effect on the OR of high triglyceride levels in men and an increasing effect in women. A Korean cross-sectional study also reported that elevated triglyceride levels were more prevalent among women than in men aged ≥ 60 years [12], which is consistent with our findings. Dysregulation of hormonal homeostasis after menopause in women is believed to cause decreased HDL-C levels and abdominal obesity [8], possibly resulting in a high prevalence of low HDL-C levels at ≥ 60 years.

High blood pressure and fasting glucose levels were significantly more prevalent among men in all age groups, with a significant increase in prevalence with age. This finding is consistent with the results of several studies, which have indicated that blood pressure increases in men beginning in adolescence, whereas in women, blood pressure increases with a decrease in hormone levels after menopause [11]. In addition, the increase in the ORs for high blood pressure and high fasting glucose levels with age was significant in both men and women. The effect of age was particularly large in women, and the increase in the OR of high fasting glucose levels was the largest among the ORs of MetS components. In Japan, where the population is expected to age further in the future, high blood pressure and high fasting glucose levels, the ORs of which increase significantly with age, are the primary components that significantly impact the prevalence of MetS. Therefore, it is essential to implement lifestyle interventions that emphasize slow eating and moderate alcohol consumption.

### Sex difference in MetS

Increase in the ORs for JIS and IDF criteria-based MetS with age in women was greater than that in men, and the prevalence in women was significantly higher than that in men in the age group > 55 years. In contrast, the prevalence of NWC criteria-based MetS, which does not include central obesity as a criterion for MetS, was significantly higher in men than in women across all age groups, and the ratio of the prevalence of NWC criteria-based MetS to that of JIS criteria-based MetS in men was approximately three times higher than that in women. The first reason for the higher prevalence of JIS and IDF criteria-based MetS in women in the older age groups is that the ORs of central obesity and high triglyceride levels increase with age in women; in contrast, they do not increase with age in men. Second, the ORs for high blood pressure and high fasting glucose levels increased with age in both men and women; however, the increase was greater in women. Meanwhile, because the NWC criteria do not include central obesity, its prevalence is higher in men in all age groups. The prevalence of NWC criteria-based MetS in men with age was high, possibly because of the increasing effect of aging on the ORs for high blood pressure and high fasting glucose. Therefore, the sex difference in the association between age and MetS prevalence may be due to the combined effects of sex differences in the association between age and MetS components. Other Asian studies using MetS criteria similar to the JIS and IDF criteria also reported a reversal of prevalence in men and women after 60 years, which is almost consistent with our findings [12–14].

### Toward the prevention of MetS and CVD

The current diagnostic criteria for MetS in Japan, as of 2022, consider the presence of central obesity a prerequisite. According to our findings, the proportion of MetS patients without central obesity who met the NWC criteria among those with MetS according to the JIS criteria was not negligible. Especially among men, the proportion increased considerably with age, from approximately 20% in the 40–45-year age group to approximately 50% in the 70–75-year age group. The effect of aging on the OR for NWC criteria-based MetS was significant in both men and women, with a larger effect in women. A study examining combinations of the prevalence of MetS components among 66-year-old Koreans found that the most common combinations among patients with three of the five MetS components were high triglyceride, high blood pressure, and high fasting glucose levels: a combination without central obesity [10]. In addition, several Japanese studies have reported that MetS screening criteria without central obesity had a higher hazard ratio for CVD [27–29, 50, 51]. In Japan, the incidence and mortality of cerebrovascular disease are higher than those of ischemic heart disease [52], and the incidence of CVD is higher among those with high blood pressure, although the proportions of central obesity and dyslipidemia are lower than those in the United States and Europe [3, 51]. Therefore, applying the MetS criteria with central obesity as a prerequisite implies that a large proportion of older Japanese men are at high risk for CVD but are not diagnosed with MetS. In Japan, patients not diagnosed with MetS do not receive active health guidance on lifestyle habits, such as eating habits; therefore, health guidance for CVD prevention may not be adequately provided. Furthermore, the prevalence of MetS varies greatly depending on the criteria and cut-off points used [28]; therefore, it is necessary to use criteria that can more accurately predict the risk of CVD according to the pathological condition and age of the individual.

The dependency rate of the older adults in Japan has reached over 28% by 2021 [53]. Many developed countries have aging populations; however, Japan is one of the most aging societies in the world, and health management for the older population is of great importance. The government has set an extension of healthy life expectancy as a goal of its healthcare policy, aiming to control the increasing burden of healthcare resources on the working-age population due to the rapid increase in the older population. Our study showed that the current MetS screening criteria are not optimal for such a goal, and that there is a high prevalence of MetS components without central obesity, especially in older men. Therefore, it is necessary to adopt the JIS or similar MetS screening criteria suitable for CVD prevention and to identify and intervene in the early stages of the disease.

### Limitations

The cohort used in this study was population-based. However, it covered most of the older population [30]; therefore, further studies using cohorts of older adults from other regions of Japan are required. In addition, although the follow-up period of this study was 6 years, it is necessary to examine the age effect over a longer period using individual-level data with a longer follow-up. Furthermore, because this study did not analyze the onset of CVD, it is necessary to examine how the pathological conditions of MetS are linked to CVD.

## Conclusions

This study was conducted using individual-level longitudinal data from 161,735 middle-aged and older Japanese participants. Overall, the increase in prevalence and ORs with age was greater in women than in men. Therefore, in Japan, where the population is aging, it is necessary to implement healthcare policies that consider sex-related differences. Furthermore, we found that a large proportion of older men with MetS according to the JIS criteria were free of central obesity. Our findings suggest the importance of adopting JIS or similar criteria that do not precondition central obesity in Japan.

## Supplementary Information

**Additional file1:** **Supplementary Table S1.** Lifestyle-related questionnaire. **Supplementary Table S2.** Individual- and regional- level intraclass correlation coefficients. **Supplementary Table S3.** Participants’ characteristics by sex and year of checkup. **Supplementary Table S4.** Prevalence of MetS and MetS components by sex and age in 2012 and 2017.

**Additional file2:**.

## Data Availability

The data that support the findings of this study are available from the Shizuoka Prefecture government in Japan, but restrictions apply to the availability of these data, which we used under license for the current study, and so are not publicly available. However, data may be available from the authors (YH: yuji.hiramatsu.pari@gmail.com) upon reasonable request and with permission from the Shizuoka prefecture government in Japan (Shizuoka prefecture government in Japan: https://www.pref.shizuoka.jp/governor/index_en.html).

## Abbreviations

MetS: Metabolic syndrome
JIS: Joint Interim Statement
IDF: International Diabetes Federation
CVD: Cardiovascular disease
NHI: National Health Insurance
WC: Waist circumference
HDL-C: High-density lipoprotein cholesterol
NWC: Not-involving waist circumference
ICC: Intraclass correlation coefficients
OR: Odds ratio
