# Supplementary material for: Differences in the components of metabolic syndrome by age and sex: a cross-sectional and longitudinal analysis of a cohort of middle-aged and older Japanese adults
Source: BMC Geriatr. 2023 Jul 17;23:438. doi: 10.1186/s12877-023-04145-0 (PMC10353138; doi:10.1186/s12877-023-04145-0)
Supplement: Supplementary file 1 — Additional file1: Supplementary Table S1. Lifestyle-related questionnaire. Supplementary Table S2. Individual- and regional- level intraclass correlation coefficients. Supplementary Table S3. Participants’ characteristics by sex and year of checkup. Supplementary Table S4. Prevalence of MetS and MetS components by sex and age in 2012 and 2017. [file 12877_2023_4145_MOESM1_ESM.docx]

# Supplementary Table S1. Lifestyle-related questionnaire

| No. | Questionnaire | Description | Answer category |
| --- | --- | --- | --- |
| Q1 | Smoking | Regular cigarette smoking. | Yes/No |
| Q2 | Exercise habits | Exercising more than 30 minutes/day for at least 2 days a week for at least 1 year. | Yes/No |
| Q3 | Physical activity | Walking or equivalent physical activity in daily life for at least 1 hour/day. | Yes/No |
| Q4 | Walking speed | Faster walking speed compared to a same-age person of the same sex. | Yes/No |
| Q5 | Eating rate | Eating rate is fast, normal, or slow compared with other people. | Fast/Normal/Slow |
| Q6 | Skipping breakfast | Skipping breakfast at least three times a week. | Yes/No |
| Q7 | Late-night dinner | Eating dinner within 2 hours before bedtime at least 3 times a week. | Yes/No |
| Q8 | Snacking | Eating a snack after dinner at least three times a week. | Yes/No |
| Q9 | Alcohol consumption frequency | Drinking alcohol every day, sometimes, or rarely. | Every day/Sometimes/Rarely |
| Q10 | Alcohol consumption | Drinking Japanese alcoholic beverages <180, 180–360, 360–540, or >540 mL/day (180 mL of a Japanese alcoholic beverage contains approximately 20 g of ethanol). | <180/180–360/360–540/>540 mL |
| Q11 | Sleeping | Getting enough rest through sleep. | Yes/No |

# Supplementary Table S2. Individual- and regional- level intraclass correlation coefficients

|  | Individual-level ICC | |  | Regional-level ICC | |
| --- | --- | --- | --- | --- | --- |
|  | Men | Women |  | Men | Women |
| Central obesity | 0.984 | 0.813 |  | 0.006 | 0.006 |
| High blood pressure | 0.613 | 0.639 |  | 0.020 | 0.012 |
| High triglyceride level | 0.742 | 0.954 |  | 0.008 | 0.012 |
| Low HDL-C level | 0.976 | 0.978 |  | 0.000 | 0.000 |
| High fasting glucose level | 0.729 | 0.713 |  | 0.023 | 0.023 |
|  |  |  |  |  |  |
| JIS | 0.954 | 0.947 |  | 0.003 | 0.001 |
| IDF | 0.976 | 0.953 |  | 0.006 | 0.001 |
| NWC | 0.956 | 0.966 |  | 0.003 | 0.001 |
| ICC: intraclass correlation coefficient; HDL-C: high-density lipoprotein cholesterol; JIS: Joint Interim Statement; IDF: International Diabetes Federation; NWC: not-involving waist circumference | | | | | |

# Supplementary Table S3. Participants’ characteristics by sex and year of checkup

|  |  |  | 2012 | | |  | 2013 | | |  | 2014 | | |
| --- | --- | --- | --- | --- | --- | --- | --- | --- | --- | --- | --- | --- | --- |
| Variable/Outcome | Unit/Category |  | Men (N=69,643) | Women (N=92,092) | *P-*value^†^ |  | Men | Women | *P-*value^†^ |  | Men | Women | *P-*value^†^ |
| Age | Years |  | 62.4 (6.9) | 62.8 (6.0) | <0.001 |  | 63.4 (6.9) | 63.8 (6.0) | <0.001 |  | 64.4 (6.9) | 64.8 (6.0) | <0.001 |
| Regional cluster^‡^ |  |  |  |  | <0.001 |  |  |  |  |  |  |  |  |
|  | Cluster 1 |  | 11.2% | 11.6% |  |  |  |  |  |  |  |  |  |
|  | Cluster 2 |  | 3.5% | 3.5% |  |  |  |  |  |  |  |  |  |
|  | Cluster 3 |  | 21.0% | 19.5% |  |  |  |  |  |  |  |  |  |
|  | Cluster 4 |  | 17.0% | 16.8% |  |  |  |  |  |  |  |  |  |
|  | Cluster 5 |  | 15.5% | 15.5% |  |  |  |  |  |  |  |  |  |
|  | Cluster 6 |  | 12.4% | 14.0% |  |  |  |  |  |  |  |  |  |
|  | Cluster 7 |  | 19.4% | 19.1% |  |  |  |  |  |  |  |  |  |
| WC | cm |  | 84.1 (8.4) | 80.0 (9.5) | <0.001 |  | 84.2 (8.5) | 80.0 (9.5) | <0.001 |  | 84.2 (8.5) | 80.1 (9.6) | <0.001 |
| Systolic blood pressure | mmHg |  | 129.1 (16.6) | 125.9 (16.8) | <0.001 |  | 129.1 (16.4) | 126.1 (16.8) | <0.001 |  | 129.6 (16.3) | 126.5 (16.7) | <0.001 |
| Diastolic blood pressure | mmHg |  | 78.4 (11.0) | 74.4 (10.7) | <0.001 |  | 78.1 (11.0) | 74.3 (10.6) | <0.001 |  | 77.9 (10.9) | 74.2 (10.6) | <0.001 |
| Triglyceride level | mg/dL |  | 131.6 (94.0) | 101.6 (60.1) | <0.001 |  | 130.5 (93.0) | 102.4 (60.7) | <0.001 |  | 128.8 (92.4) | 101.8 (58.9) | <0.001 |
| HDL-C level | mg/dL |  | 58.0 (15.3) | 68.0 (16.0) | <0.001 |  | 58.0 (15.2) | 68.1 (16.0) | <0.001 |  | 58.4 (15.5) | 68.5 (16.2) | <0.001 |
| HbA1c level | % |  | 5.7 (0.7) | 5.6 (0.5) | <0.001 |  | 5.7 (0.7) | 5.7 (0.5) | <0.001 |  | 5.8 (0.7) | 5.7 (0.5) | <0.001 |
| Fasting plasma glucose | mg/dL |  | 101.2 (22.1) | 94.0 (15.3) | <0.001 |  | 101.2 (21.3) | 94.1 (15.8) | <0.001 |  | 101.4 (20.9) | 94.4 (15.6) | <0.001 |
| Smoking^¶^ | Yes |  | 24.9% | 5.4% | <0.001 |  | 24.1% | 5.3% | <0.001 |  | 23.2% | 5.1% | <0.001 |
|  | No |  | 75.1% | 94.6% |  |  | 75.9% | 94.7% |  |  | 76.8% | 94.9% |  |
| Exercise habits^¶^ | Yes |  | 41.7% | 36.9% | <0.001 |  | 43.6% | 38.9% | <0.001 |  | 44.8% | 40.5% | <0.001 |
|  | No |  | 58.3% | 63.1% |  |  | 56.4% | 61.1% |  |  | 55.2% | 59.5% |  |
| Physical activity^¶^ | Yes |  | 50.1% | 47.9% | <0.001 |  | 51.2% | 50.2% | 0.003 |  | 51.9% | 51.3% | 0.061 |
|  | No |  | 49.9% | 52.1% |  |  | 48.8% | 49.8% |  |  | 48.1% | 48.7% |  |
| Walking speed^¶^ | Yes |  | 48.7% | 45.0% | <0.001 |  | 48.6% | 45.7% | <0.001 |  | 49.3% | 46.0% | <0.001 |
|  | No |  | 51.3% | 55.0% |  |  | 51.4% | 54.3% |  |  | 50.7% | 54.0% |  |
| Eating rate^¶^ |  |  |  |  | <0.001 |  |  |  | <0.001 |  |  |  | <0.001 |
|  | Fast |  | 29.8% | 22.8% |  |  | 29.0% | 22.1% |  |  | 28.1% | 21.4% |  |
|  | Normal |  | 62.9% | 69.8% |  |  | 63.9% | 70.8% |  |  | 64.5% | 71.3% |  |
|  | Slow |  | 7.3% | 7.4% |  |  | 7.1% | 7.1% |  |  | 7.4% | 7.3% |  |
| Skipping breakfast^¶^ | Yes |  | 8.7% | 4.9% | <0.001 |  | 8.4% | 4.9% | <0.001 |  | 8.1% | 4.8% | <0.001 |
|  | No |  | 91.3% | 95.1% |  |  | 91.6% | 95.1% |  |  | 91.9% | 95.2% |  |
| Late-night dinner^¶^ | Yes |  | 16.5% | 7.1% | <0.001 |  | 16.2% | 6.9% | <0.001 |  | 16.1% | 6.9% | <0.001 |
|  | No |  | 83.5% | 92.9% |  |  | 83.8% | 93.1% |  |  | 83.9% | 93.1% |  |
| Snacking^¶^ | Yes |  | 11.4% | 11.4% | >0.9 |  | 10.6% | 11.0% | 0.11 |  | 10.6% | 10.5% | >0.9 |
|  | No |  | 88.6% | 88.6% |  |  | 89.4% | 89.0% |  |  | 89.4% | 89.5% |  |
| Alcohol consumption frequency^¶^ |  |  |  |  | <0.001 |  |  |  | <0.001 |  |  |  | <0.001 |
|  | Every day |  | 45.0% | 8.2% |  |  | 44.7% | 8.2% |  |  | 44.5% | 8.2% |  |
|  | Sometimes |  | 23.6% | 20.7% |  |  | 23.1% | 20.1% |  |  | 22.7% | 19.7% |  |
|  | Rarely |  | 31.4% | 71.1% |  |  | 32.2% | 71.7% |  |  | 32.8% | 72.1% |  |
| Alcohol consumption^¶^ |  |  |  |  | <0.001 |  |  |  | <0.001 |  |  |  | <0.001 |
|  | <180 mL |  | 46.7% | 88.2% |  |  | 47.0% | 88.3% |  |  | 47.6% | 88.6% |  |
|  | 180–360 mL |  | 32.7% | 9.1% |  |  | 32.5% | 9.0% |  |  | 32.3% | 8.9% |  |
|  | 360–540 mL |  | 16.3% | 2.1% |  |  | 16.5% | 2.2% |  |  | 16.1% | 2.0% |  |
|  | >540 mL |  | 4.3% | 0.6% |  |  | 4.0% | 0.5% |  |  | 4.0% | 0.5% |  |
| Sleeping^¶^ | Yes |  | 79.3% | 74.2% | <0.001 |  | 79.1% | 74.5% | <0.001 |  | 79.3% | 74.9% | <0.001 |
|  | No |  | 20.7% | 25.8% |  |  | 20.9% | 25.5% |  |  | 20.7% | 25.1% |  |
| Mean (SD); % | | | | | | | | | | | | | |
| † Wilcoxon rank sum test; Chi-square test | | | | | | | | | | | | | |
| ‡ The proportion of clusters does not change with checkup year. | | | | | | | | | | | | | |
| ¶ See Supplementary Table S1 for the definitions of each lifestyle-related variables. | | | | | | | | | | | | | |
| WC: waist circumference; HDL-C: high-density lipoprotein cholesterol; HbA1c: hemoglobin A1c | | | | | | | | | | | | | |

(continued)

Supplementary Table S3. (continued)

|  |  |  | 2015 | | |  | 2016 | | |  | 2017 | | |
| --- | --- | --- | --- | --- | --- | --- | --- | --- | --- | --- | --- | --- | --- |
| Variable/Outcome | Unit/Category |  | Men | Women | *P*-value† |  | Men | Women | *P*-value† |  | Men | Women | *P*-value† |
| Age | Years |  | 65.4 (6.9) | 65.8 (6.0) | <0.001 |  | 66.4 (6.9) | 66.8 (6.0) | <0.001 |  | 67.4 (6.9) | 67.8 (6.0) | <0.001 |
| Regional cluster^‡^ |  |  |  |  |  |  |  |  |  |  |  |  |  |
|  | Cluster 1 |  |  |  |  |  |  |  |  |  |  |  |  |
|  | Cluster 2 |  |  |  |  |  |  |  |  |  |  |  |  |
|  | Cluster 3 |  |  |  |  |  |  |  |  |  |  |  |  |
|  | Cluster 4 |  |  |  |  |  |  |  |  |  |  |  |  |
|  | Cluster 5 |  |  |  |  |  |  |  |  |  |  |  |  |
|  | Cluster 6 |  |  |  |  |  |  |  |  |  |  |  |  |
|  | Cluster 7 |  |  |  |  |  |  |  |  |  |  |  |  |
| WC | cm |  | 84.3 (8.6) | 80.2 (9.6) | <0.001 |  | 84.4 (8.7) | 80.3 (9.7) | <0.001 |  | 84.6 (8.8) | 80.4 (9.8) | <0.001 |
| Systolic blood pressure | mmHg |  | 129.6 (16.3) | 126.7 (16.7) | <0.001 |  | 129.5 (16.2) | 126.8 (16.6) | <0.001 |  | 130.1 (16.4) | 127.8 (16.9) | <0.001 |
| Diastolic blood pressure | mmHg |  | 77.5 (10.9) | 73.8 (10.5) | <0.001 |  | 76.9 (10.9) | 73.4 (10.5) | <0.001 |  | 77.2 (11.0) | 73.8 (10.6) | <0.001 |
| Triglyceride level | mg/dL |  | 127.3 (88.8) | 101.7 (58.3) | <0.001 |  | 126.4 (87.8) | 102.2 (59.1) | <0.001 |  | 126.8 (89.5) | 102.8 (59.9) | <0.001 |
| HDL-C level | mg/dL |  | 58.0 (15.3) | 68.2 (16.2) | <0.001 |  | 58.2 (15.4) | 68.4 (16.2) | <0.001 |  | 58.3 (15.4) | 68.8 (16.3) | <0.001 |
| HbA1c level | % |  | 5.8 (0.7) | 5.7 (0.5) | <0.001 |  | 5.8 (0.7) | 5.7 (0.5) | <0.001 |  | 5.8 (0.7) | 5.7 (0.5) | <0.001 |
| Fasting plasma glucose | mg/dL |  | 101.5 (21.5) | 94.5 (15.2) | <0.001 |  | 102.1 (21.4) | 95.0 (15.8) | <0.001 |  | 102.1 (21.1) | 95.2 (15.9) | <0.001 |
| Smoking^¶^ | Yes |  | 22.8% | 5.0% | <0.001 |  | 21.8% | 4.8% | <0.001 |  | 20.8% | 4.7% | <0.001 |
|  | No |  | 77.2% | 95.0% |  |  | 78.2% | 95.2% |  |  | 79.2% | 95.3% |  |
| Exercise habits^¶^ | Yes |  | 44.8% | 40.9% | <0.001 |  | 45.7% | 42.0% | <0.001 |  | 45.3% | 42.2% | <0.001 |
|  | No |  | 55.2% | 59.1% |  |  | 54.3% | 58.0% |  |  | 54.7% | 57.8% |  |
| Physical activity^¶^ | Yes |  | 52.4% | 51.8% | 0.039 |  | 52.4% | 52.5% | 0.70 |  | 52.7% | 52.6% | 0.8 |
|  | No |  | 47.6% | 48.2% |  |  | 47.6% | 47.5% |  |  | 47.3% | 47.4% |  |
| Walking speed^¶^ | Yes |  | 49.2% | 46.4% | <0.001 |  | 48.5% | 46.3% | <0.001 |  | 48.2% | 46.0% | <0.001 |
|  | No |  | 50.8% | 53.6% |  |  | 51.5% | 53.7% |  |  | 51.8% | 54.0% |  |
| Eating rate^¶^ |  |  |  |  | <0.001 |  |  |  | <0.001 |  |  |  | <0.001 |
|  | Fast |  | 27.4% | 21.0% |  |  | 26.6% | 20.7% |  |  | 26.4% | 20.5% |  |
|  | Normal |  | 65.0% | 71.7% |  |  | 65.6% | 71.9% |  |  | 65.8% | 72.1% |  |
|  | Slow |  | 7.6% | 7.3% |  |  | 7.8% | 7.4% |  |  | 7.8% | 7.4% |  |
| Skipping breakfast^¶^ | Yes |  | 8.1% | 4.8% | <0.001 |  | 8.0% | 4.8% | <0.001 |  | 8.0% | 4.7% | <0.001 |
|  | No |  | 91.9% | 95.2% |  |  | 92.0% | 95.2% |  |  | 92.0% | 95.3% |  |
| Late-night dinner^¶^ | Yes |  | 16.0% | 6.9% | <0.001 |  | 15.6% | 6.8% | <0.001 |  | 15.6% | 6.8% | <0.001 |
|  | No |  | 84.0% | 93.1% |  |  | 84.4% | 93.2% |  |  | 84.4% | 93.2% |  |
| Snacking^¶^ | Yes |  | 10.0% | 10.4% | 0.059 |  | 10.0% | 9.8% | 0.200 |  | 9.8% | 9.5% | 0.130 |
|  | No |  | 90.0% | 89.6% |  |  | 90.0% | 90.2% |  |  | 90.2% | 90.5% |  |
| Alcohol consumption frequency^¶^ |  |  |  |  | <0.001 |  |  |  | <0.001 |  |  |  | <0.001 |
|  | Every day |  | 44.3% | 8.2% |  |  | 43.8% | 8.2% |  |  | 43.4% | 8.3% |  |
|  | Sometimes |  | 22.5% | 19.4% |  |  | 22.6% | 19.0% |  |  | 22.2% | 18.7% |  |
|  | Rarely |  | 33.2% | 72.4% |  |  | 33.6% | 72.8% |  |  | 34.4% | 73.0% |  |
| Alcohol consumption^¶^ |  |  |  |  | <0.001 |  |  |  | <0.001 |  |  |  | <0.001 |
|  | <180 mL |  | 47.6% | 88.5% |  |  | 48.6% | 88.8% |  |  | 47.8% | 87.7% |  |
|  | 180–360 mL |  | 32.3% | 8.8% |  |  | 32.1% | 8.8% |  |  | 33.0% | 9.6% |  |
|  | 360–540 mL |  | 16.1% | 2.1% |  |  | 15.7% | 1.9% |  |  | 15.7% | 2.1% |  |
|  | >540 mL |  | 4.0% | 0.6% |  |  | 3.6% | 0.5% |  |  | 3.5% | 0.6% |  |
| Sleeping^¶^ | Yes |  | 78.8% | 74.4% | <0.001 |  | 78.1% | 74.2% | <0.001 |  | 77.5% | 73.3% | <0.001 |
|  | No |  | 21.2% | 25.6% |  |  | 21.9% | 25.8% |  |  | 22.5% | 26.7% |  |

**Supplementary Table S4. Prevalence of MetS and MetS components by sex and age in 2012 and 2017**

| As of 2012 |  |  | 40–45 years | | |  | 45–50 years | | |  | 50–55 years | | |  | 55–60 years | | |  | 60–65 years | | |  | 65–70 years | | |
| --- | --- | --- | --- | --- | --- | --- | --- | --- | --- | --- | --- | --- | --- | --- | --- | --- | --- | --- | --- | --- | --- | --- | --- | --- | --- |
|  |  |  | Men (N=3,468) | Women (N=3,008) | *P*-value^†^ |  | Men (N=4,245) | Women (N=3,902) | *P*-value^†^ |  | Men (N=4,590) | Women (N=5,371) | *P*-value^†^ |  | Men (N=6,761) | Women (N=10,415) | *P*-value^†^ |  | Men (N=19,754) | Women (N=30,655) | *P*-value^†^ |  | Men (N=30,825) | Women (N=38,741) | *P*-value^†^ |
| MetS components |  |  |  |  |  |  |  |  |  |  |  |  |  |  |  |  |  |  |  |  |  |  |  |  |  |
|  | Central obesity |  | 24.6% | 32.5% | <0.001 |  | 25.9% | 38.3% | <0.001 |  | 25.6% | 41.2% | <0.001 |  | 25.0% | 46.7% | <0.001 |  | 23.3% | 49.6% | <0.001 |  | 21.0% | 53.0% | <0.001 |
|  | High blood pressure |  | 30.9% | 14.9% | <0.001 |  | 40.4% | 24.2% | <0.001 |  | 47.9% | 32.1% | <0.001 |  | 57.7% | 42.8% | <0.001 |  | 65.4% | 52.2% | <0.001 |  | 69.7% | 60.7% | <0.001 |
|  | High triglyceride level |  | 35.5% | 9.2% | <0.001 |  | 37.6% | 13.1% | <0.001 |  | 38.4% | 18.2% | <0.001 |  | 39.1% | 26.6% | <0.001 |  | 38.8% | 34.3% | <0.001 |  | **38.1%** | **39.2%** | **0.018** |
|  | Low HDL-C level |  | 12.2% | 11.5% | 0.6 |  | 12.0% | 12.3% | 0.8 |  | 10.1% | 9.0% | 0.2 |  | 10.5% | 11.0% | 0.4 |  | **9.6%** | **13.0%** | **<0.001** |  | 9.7% | 13.9% | <0.001 |
|  | High fasting glucose level |  | 26.8% | 15.1% | <0.001 |  | 34.3% | 20.7% | <0.001 |  | 41.2% | 29.3% | <0.001 |  | 48.6% | 40.8% | <0.001 |  | 53.8% | 47.0% | <0.001 |  | 56.5% | 49.9% | <0.001 |
| MetS |  |  |  |  |  |  |  |  |  |  |  |  |  |  |  |  |  |  |  |  |  |  |  |  |  |
|  | JIS |  | 17.3% | 8.8% | <0.001 |  | 23.2% | 13.7% | <0.001 |  | 24.7% | 16.6% | <0.001 |  | 29.1% | 25.4% | <0.001 |  | **30.5%** | **33.6%** | **<0.001** |  | 30.7% | 39.4% | <0.001 |
|  | IDF |  | 13.6% | 8.5% | <0.001 |  | 16.8% | 13.0% | <0.001 |  | 17.5% | 15.5% | 0.042 |  | **18.3%** | **22.6%** | **<0.001** |  | 17.4% | 28.7% | <0.001 |  | 16.1% | 33.3% | <0.001 |
|  | NWC |  | 3.7% | 0.3% | <0.001 |  | 6.4% | 0.6% | <0.001 |  | 7.2% | 1.1% | <0.001 |  | 10.7% | 2.8% | <0.001 |  | 13.1% | 4.9% | <0.001 |  | 14.7% | 6.1% | <0.001 |
|  |  |  |  |  |  |  |  |  |  |  |  |  |  |  |  |  |  |  |  |  |  |  |  |  |  |
|  | IDF/JIS |  | 78.6% | 96.6% |  |  | 72.4% | 94.9% |  |  | 70.9% | 93.4% |  |  | 62.9% | 89.0% |  |  | 57.0% | 85.4% |  |  | 52.4% | 84.5% |  |
|  | NWC/JIS |  | 21.4% | 3.4% |  |  | 27.6% | 5.1% |  |  | 29.1% | 6.6% |  |  | 37.1% | 11.0% |  |  | 43.0% | 14.6% |  |  | 47.6% | 15.5% |  |
|  |  |  |  |  |  |  |  |  |  |  |  |  |  |  |  |  |  |  |  |  |  |  |  |  |  |
| As of 2017 |  |  | 45–50 years | | |  | 50–55 years | | |  | 55–60 years | | |  | 60–65 years | | |  | 65–70 years | | |  | 70–75 years | | |
|  |  |  | Men (N=3,468) | Women (N=3,008) | *P*-value^†^ |  | Men (N=4,245) | Women (N=3,902) | *P*-value^†^ |  | Men (N=4,590) | Women (N=5,371) | *P*-value^†^ |  | Men (N=6,761) | Women (N=10,415) | *P*-value^†^ |  | Men (N=19,754) | Women (N=30,655) | *P*-value^†^ |  | Men (N=30,825) | Women (N=38,741) | *P*-value^†^ |
| MetS components |  |  |  |  |  |  |  |  |  |  |  |  |  |  |  |  |  |  |  |  |  |  |  |  |  |
|  | Central obesity |  | 28.8% | 37.3% | <0.001 |  | 29.5% | 40.8% | <0.001 |  | 28.5% | 43.8% | <0.001 |  | 28.4% | 49.2% | <0.001 |  | 25.1% | 52.2% | <0.001 |  | 23.2% | 54.3% | <0.001 |
|  | High blood pressure |  | 40.1% | 21.7% | <0.001 |  | 47.8% | 31.0% | <0.001 |  | 56.5% | 39.0% | <0.001 |  | 65.1% | 50.0% | <0.001 |  | 71.1% | 59.2% | <0.001 |  | 74.7% | 68.0% | <0.001 |
|  | High triglyceride level |  | 37.3% | 12.3% | <0.001 |  | 39.3% | 17.9% | <0.001 |  | 42.1% | 25.6% | <0.001 |  | 40.3% | 34.2% | <0.001 |  | 41.9% | 41.6% | 0.6 |  | **40.9%** | **47.2%** | **<0.001** |
|  | Low HDL-C level |  | 11.2% | 8.8% | 0.019 |  | 10.4% | 10.1% | 0.7 |  | 10.4% | 9.3% | 0.120 |  | **9.1%** | **10.2%** | **0.041** |  | 9.2% | 12.3% | <0.001 |  | 10.0% | 13.3% | <0.001 |
|  | High fasting glucose level |  | 44.0% | 29.2% | <0.001 |  | 50.6% | 40.3% | <0.001 |  | 57.7% | 49.5% | <0.001 |  | 63.3% | 58.0% | <0.001 |  | 66.8% | 62.5% | <0.001 |  | 68.9% | 64.9% | <0.001 |
| MetS |  |  |  |  |  |  |  |  |  |  |  |  |  |  |  |  |  |  |  |  |  |  |  |  |  |
|  | JIS |  | 25.6% | 12.6% | <0.001 |  | 29.6% | 19.3% | <0.001 |  | 32.4% | 25.3% | <0.001 |  | 34.6% | 34.9% | 0.7 |  | **37.1%** | **43.1%** | **<0.001** |  | 37.5% | 49.6% | <0.001 |
|  | IDF |  | 19.6% | 12.0% | <0.001 |  | 20.8% | 17.8% | 0.008 |  | 21.4% | 22.4% | 0.3 |  | **22.3%** | **30.4%** | **<0.001** |  | 20.4% | 36.4% | <0.001 |  | 19.4% | 40.7% | <0.001 |
|  | NWC |  | 6.0% | 0.6% | <0.001 |  | 8.9% | 1.6% | <0.001 |  | 11.0% | 2.9% | <0.001 |  | 12.2% | 4.5% | <0.001 |  | 16.7% | 6.7% | <0.001 |  | 18.1% | 8.9% | <0.001 |
|  |  |  |  |  |  |  |  |  |  |  |  |  |  |  |  |  |  |  |  |  |  |  |  |  |  |
|  | IDF/JIS |  | 76.6% | 95.2% |  |  | 70.3% | 92.2% |  |  | 66.0% | 88.5% |  |  | 64.5% | 87.1% |  |  | 55.0% | 84.5% |  |  | 51.7% | 82.1% |  |
|  | NWC/JIS |  | 23.4% | 4.8% |  |  | 29.7% | 7.8% |  |  | 34.0% | 11.5% |  |  | 35.5% | 12.9% |  |  | 45.0% | 15.5% |  |  | 48.3% | 17.9% |  |
|  |  |  |  |  |  |  |  |  |  |  |  |  |  |  |  |  |  |  |  |  |  |  |  |  |  |
| † Chi-square test | | | | | | | | | | | | | | | | | | | | | | | | | |
| **Bold type: representing a significant reversal of the prevalence of MetS components and MetS between men and women.** | | | | | | | | | | | | | | | | | | | | | | | | | |
| MetS: metabolic syndrome; HDL-C: high-density lipoprotein cholesterol; JIS: Joint Interim Statement; IDF: International Diabetes Federation; NWC: not-involving waist circumference | | | | | | | | | | | | | | | | | | | | | | | | | |
