# Supplementary figures and images for: Differences in the components of metabolic syndrome by age and sex: a cross-sectional and longitudinal analysis of a cohort of middle-aged and older Japanese adults
Source: BMC Geriatr. 2023 Jul 17;23:438. doi: 10.1186/s12877-023-04145-0 (PMC10353138; doi:10.1186/s12877-023-04145-0)

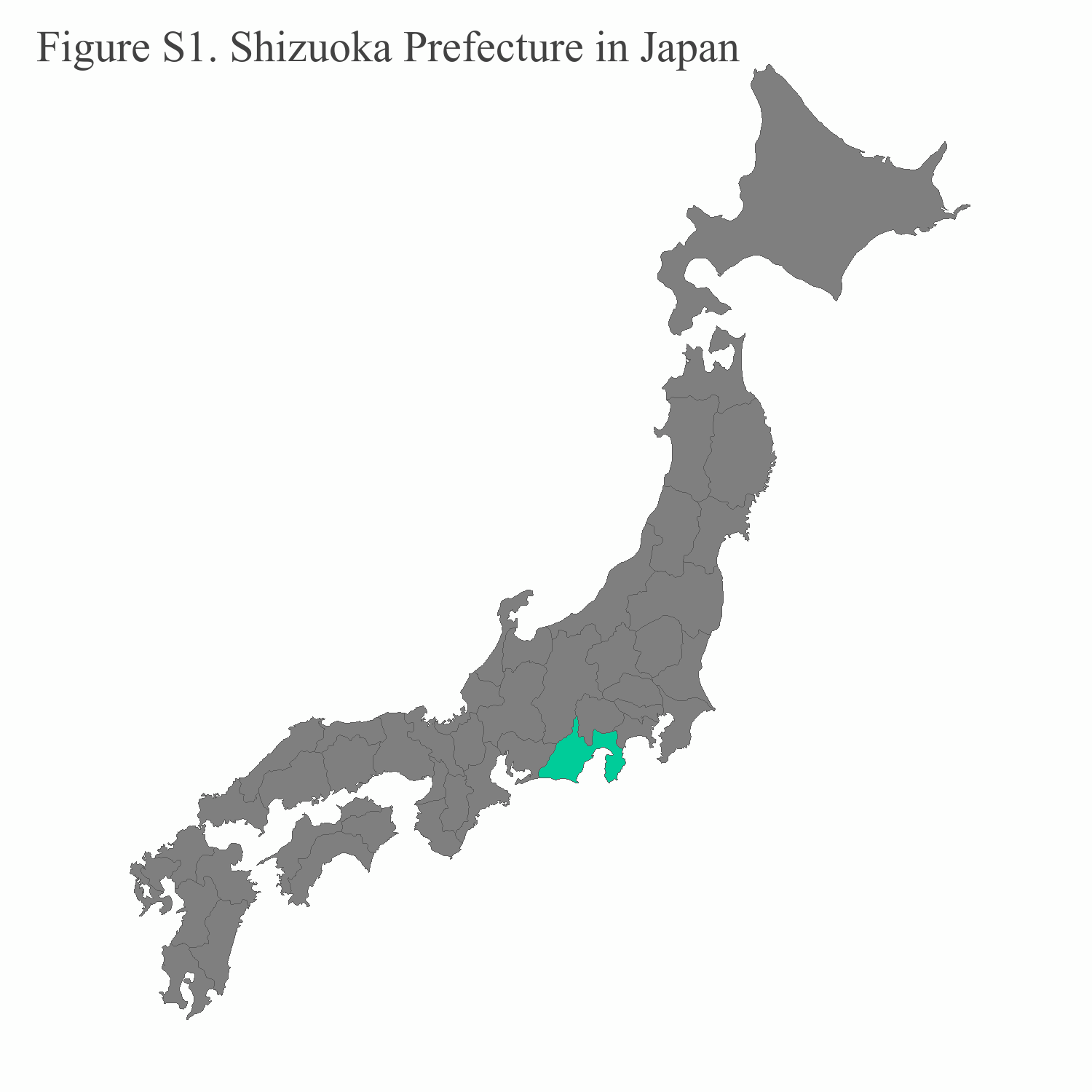

Supplement: Supplementary file 2 — Additional file2:. [file 12877_2023_4145_MOESM2_ESM.tiff]
